# Pulmonary Effects of Traumatic Brain Injury in Mice: A Gene Set Enrichment Analysis

**DOI:** 10.3390/ijms25053018

**Published:** 2024-03-05

**Authors:** Wei-Hung Chan, Shih-Ming Huang, Yi-Lin Chiu

**Affiliations:** 1 Department of Anesthesiology, Tri-Service General Hospital, National Defense Medical Center, Taipei City 114201, Taiwan; whcken@ndmctsgh.edu.tw; 2 Graduate Institute of Medical Sciences, National Defense Medical Center, Taipei City 114201, Taiwan; 3 Department of Biochemistry, National Defense Medical Center, Taipei City 114201, Taiwan; shihming@ndmctsgh.edu.tw

**Keywords:** traumatic brain injury, acute lung injury, immune cell infiltration, brain lung interaction

## Abstract

Acute lung injury occurs in 20–25% of cases following traumatic brain injury (TBI). We investigated changes in lung transcriptome expression post-TBI using animal models and bioinformatics. Employing unilateral controlled cortical impact for TBI, we conducted microarray analysis after lung acquisition, followed by gene set enrichment analysis of differentially expressed genes. Our findings indicate significant upregulation of inflammation-related genes and downregulation of nervous system genes. There was enhanced infiltration of adaptive immune cells, evidenced by positive enrichment in Lung-Th1, CD4, and CD8 T cells. Analysis using the Tabula Sapiens database revealed enrichment in lung-adventitial cells, pericytes, myofibroblasts, and fibroblasts, indicating potential effects on lung vasculature and fibrosis. Gene set enrichment analysis linked TBI to lung diseases, notably idiopathic pulmonary hypertension. A Venn diagram overlap analysis identified a common set of 20 genes, with *FOSL2* showing the most significant fold change. Additionally, we observed a significant increase in *ADRA1A*→*IL6* production post-TBI using the L1000 library. Our study highlights the impact of brain trauma on lung injury, revealing crucial gene expression changes related to immune cell infiltration, cytokine production, and potential alterations in lung vasculature and fibrosis, along with a specific spectrum of disease influence.

## 1. Introduction

Traumatic brain Injury (TBI), resulting from external forces such as falls, motor vehicle accidents, and violence, is a leading cause of disability and death among otherwise healthy adults [1]. It poses a significant public health challenge [2], with its full effects on the body yet to be thoroughly understood. Each year, more than 50 million people worldwide suffer from traumatic brain injuries, and it is estimated that approximately half of the global population will encounter at least one TBI in their lifetime [3]. TBI disproportionately affecting low- to middle-income countries [4]. Financially, TBI results in approximately $400 billion in global losses annually, placing a considerable medical burden on societies, individuals, and healthcare systems [5,6].

The broad systemic effects of TBI extend beyond the immediate neurological damage [7]. Research has shown that TBI can lead to significant dysfunction in other organs [8,9], including the kidneys [10] and lungs [11], thereby adding to the injury’s complexity and the challenge of treatment. For instance, severe TBI is associated with a high mortality rate [6,12,13] and can lead to neurological effects, such as epilepsy, as well as to non-neurological damage [7,8,14], including infection [15,16], endocrine dysfunction [14], acute lung injury (ALI) [11,17], and acute kidney injury [10], emphasizing the interconnectedness of body systems in response to TBI. The incidence of acute kidney injury can reach up to 20% [10], and the probability of acute lung injury can range between 20% to 25% [11,17]. Among these organs, the lung is particularly susceptible to inflammation caused by TBI [18]. The development of ALI following TBI can lead to mortality rates as high as 28% [17] to 38% [19], and ALI after TBI is also associated with poorer long-term neurological outcomes in survivors [19]. While strategies for lung protection exist for treating ALI or acute respiratory distress syndrome (ARDS) [20,21,22], their application in neurocritical care remains ambiguous, primarily because many of these ventilatory approaches carry a heightened risk of inducing intracranial hypertension [23]. Conversely, treatments aimed at anti-inflammation or immune modulation [24] have also been investigated [25,26] for lung treatment after TBI, since TBI may induce systemic inflammation [27] and impair lung immunity [28].

In previous studies carried out by our lab, we observed that TBI affected gene sets related to mitochondrial function in renal cells and saw a negative enrichment in gene sets linked with immune cell migration and epidermal development [29]. Despite some progress in understanding the pathophysiology of TBI, the mechanisms linking TBI to systemic organ damage, particularly lung damage, remain poorly understood [7,9,27,30,31]. Therefore, the purpose of this study was to explore the immune responses, molecular or cellular interactions, and biochemical pathways implicated in TBI-induced lung injury, employing animal models and bioinformatics methodologies. By identifying potential changes in the overall lung transcriptome expression following TBI, our research aims to uncover novel insights into the immune and molecular mechanisms at play, offering avenues for the development of targeted treatments and prevention strategies for TBI-induced lung injury.

## 2. Results

### 2.1. Gene Expression Alterations in TBI-Affected Lung Tissues

**Figure 1:**
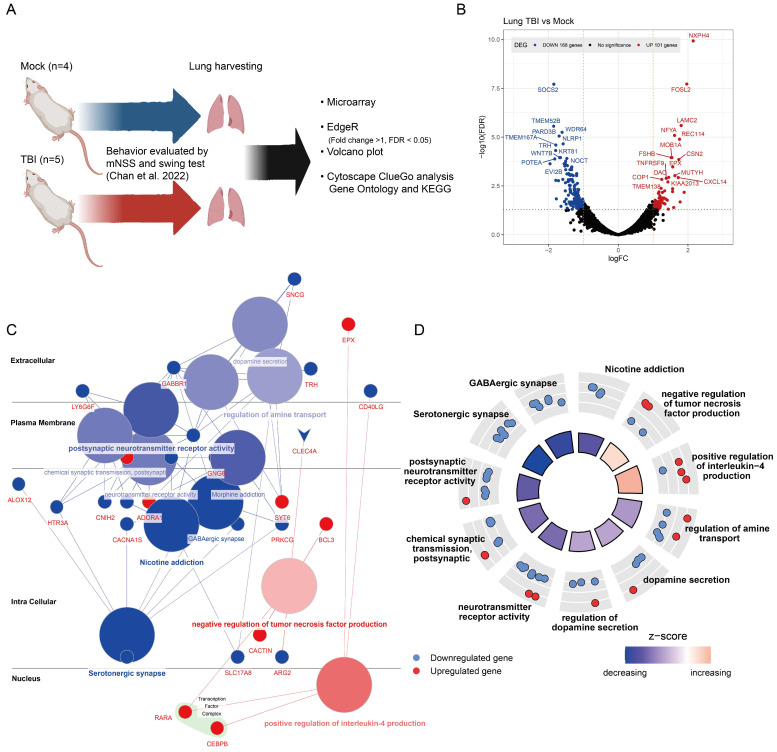
Gene expression changes in lung tissue after TBI: (**A**) mouse model schematic for TBI analysis [22]; (**B**) volcano plot showing differentially expressed genes, with upregulation in red, downregulation in blue, based on log2 Fold Change > 1 (indicated by orange vertical dashed lines) and FDR < 0.05 (indicated by red horizontal dashed lines). Top 30 significant genes are highlighted; (**C**) ClueGO visualization of gene distribution and associations in cellular structures. Upregulated and downregulated genes are marked with red and blue dots, respectively; gene sets are shown as circles, with color intensity indicating association strength; and (**D**) GOcircle plot of gene expression within gene sets, with z-scores reflecting differential expression significance.

Our previous study evaluated the potential impact of TBI on kidney tissue, as well as the effects on mouse behavior and biochemical indices. Continuing from that research, we carried out an investigation on lung tissue samples obtained from the same experiments, focusing on mRNA studies. Utilizing the EdgeR software, we analyzed differentially expressed genes (DEG), which are displayed in a volcano plot. Further analysis of gene ontology and KEGG biological pathway enrichment was performed using the Cytoscape ClueGO app, as depicted in Figure 1A. We applied a selection criterion of an absolute log2 fold change value greater than 1 and a false discovery rate (FDR) less than 0.05, based on the Benjamini–Hochberg Procedure. This selection identified 168 downregulated genes and 101 upregulated genes, as shown in Figure 1B. The upregulated genes included *NXPH4*, *FOSL2*, *LAMC2*, *NFYA*, *REC114*, and *CXCL14*, among others. Downregulated genes included *SOCS2*, *TMEM52B*, *WDR64*, *TMEM167A*, *KRT81*, and more.

A gene association analysis of these differentially expressed genes was conducted via Cytoscape, uncovering two key insights: (1) inflammation as a central response. The upregulation of genes associated with inflammation, especially those involved in ‘positive regulation of interleukin-4 production’, highlights the lung’s dynamic inflammatory response to TBI. This finding is crucial, as it indicates a protective or compensatory mechanism designed to alleviate lung injury post-TBI (Figure 1C); and (2) the neurological impact on pulmonary systems. The downregulation of genes linked to the nervous system underscores a significant, albeit indirect, effect of TBI on pulmonary neuro-signaling pathways. This decrease, particularly in genes responsible for ‘dopamine secretion’ and ‘neurotransmitter receptor activity’, prompts vital considerations regarding neuro-immune interactions and their consequences for lung function after TBI (Figure 1C).

The gene CCAAT/enhancer-binding protein β (*C/EBPβ*), related to the ‘positive regulation of interleukin-4 production’, is a transcription factor previously reported to be involved in IgG immune-complex-induced acute lung injury [32], aligning with our prior publication. Utilizing GOplot’s GOcircle functionality to display the distribution of individual enriched gene sets and associated genes revealed that the overall z-score for gene sets related to inflammation increased, indicating that most associated genes were upregulated in lung tissue following TBI. Conversely, the overall z-score for gene sets related to the nervous system decreased, suggesting a potential impact of TBI on the pulmonary nervous system (Figure 1D).

### 2.2. C/EBPβ-Associated Gene Enrichment in Post-TBI Lung Tissues and Implications for Macrophage Activation

**Figure 2:**
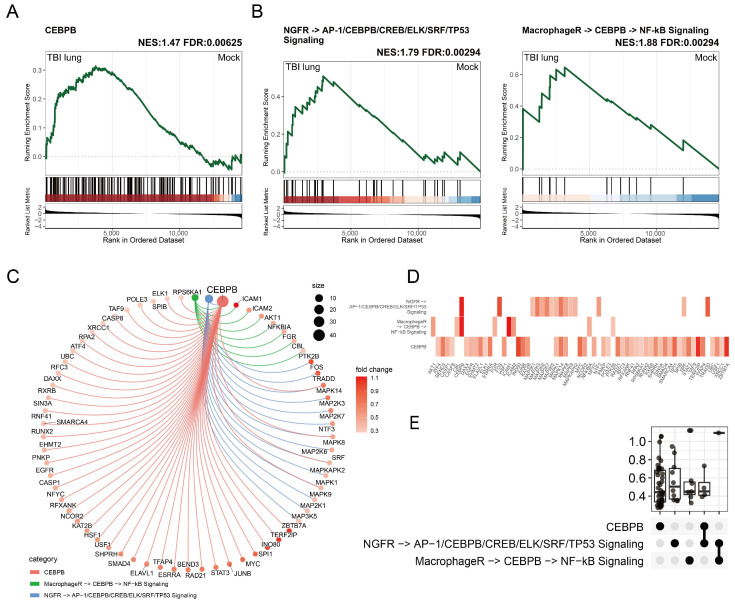
*C/EBPβ*-associated gene sets in lung tissues after TBI: role in macrophage activation: (**A**,**B**) GSEA displaying *C/EBPβ* and related gene sets; (**C**) CNET plot of key gene distributions and expressions; (**D**) heatmap of gene overlap and uniqueness in sets; and (**E**) upset plot showing connections between the *C/EBPβ* gene set and associated signaling pathways.

In the preceding section, we unveiled a notable enrichment of *C/EBPβ* within transcription factor complexes in lung tissues following TBI. Employing gene set enrichment analysis (GSEA), we discerned a significant enrichment of the *C/EBPβ* gene set in lung tissues post-TBI, with a normalized *e*nrichment *s*core (NES) of 1.47 and an FDR below 0.05 (Figure 2A). This finding highlights C/EBPβ’s influence on lung tissue recovery and inflammation management after TBI. The study was extended to examine additional gene sets, including *NGFR→AP-1/C/EBPβ/CREB/ELK/SRF/TP53* signaling and *MacrophageR→C/EBPβ→NF-kB* signaling, which exhibited significant enrichments with NES values of 1.79 and 1.88, respectively (Figure 2B). A critical insight from our analysis is the *MacrophageR→C/EBPβ→NF-kB* pathway’s association with key genes such as ICAM1, ICAM2, AKT1, NFKB1, FGR, and FBL, which unveils a crucial mechanism by which C/EBPβ may drive macrophage activation and the subsequent inflammatory response, pivotal for repair mechanisms and defense in lung tissues post-injury. Furthermore, the tight interrelation between the *C/EBPβ* gene set and the *NGFR→AP-1/C/EBPβ/CREB/ELK/SRF/TP53* signaling pathway, along with the distinctiveness of the leading-edge genes of the *MacrophageR→C/EBPβ→NF-kB* pathway from other sets, elucidates the complex network of signaling pathways activated in response to TBI. This complexity suggests that multiple, potentially overlapping mechanisms contribute to the lung tissue’s response to injury, with C/EBPβ playing a central coordinating role. The utilization of CNET analysis provided deeper insights into the distribution and expression patterns of pivotal genes (Figure 2C), enhancing our understanding of their specific roles and interactions within the lung tissue’s response to TBI. The minimal overlap among genes within the highlighted pathways, as demonstrated by the heatmap, and the detailed connections revealed by the upset plot (Figure 2D), emphasize the uniqueness and importance of each gene set in the post-TBI pulmonary environment. These observations not only reinforce the significant role of C/EBPβ in influencing macrophage behavior and the immune response following TBI but also offer a broader perspective on the molecular mechanisms at play. This comprehensive understanding provides a foundation for targeted therapeutic strategies aimed at mitigating lung injury and promoting recovery in TBI patients.

### 2.3. Immune Response Modulation in Post-TBI Lung Tissue: Insights from x-Cell and GSEA Analyses

**Figure 3:**
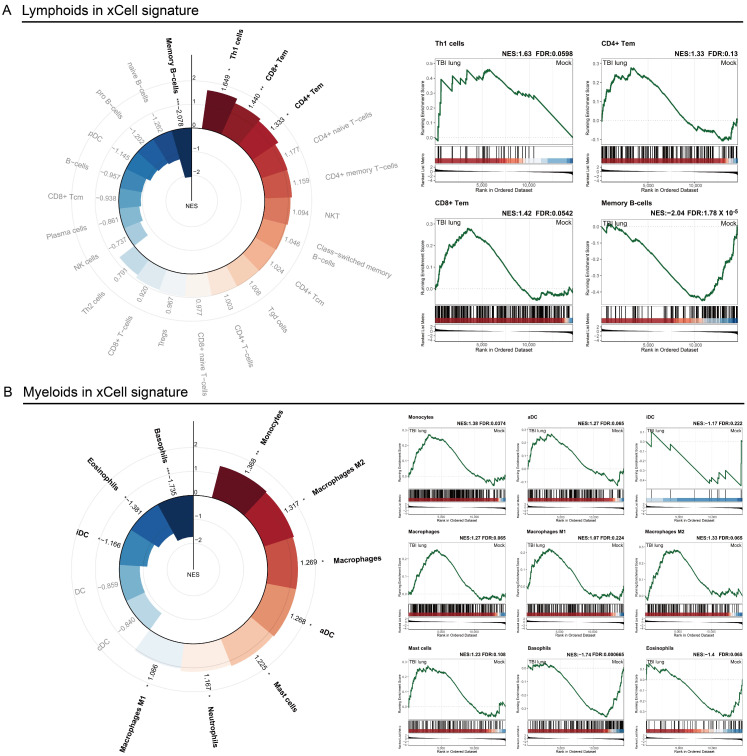
Lymphoid and myeloid cellular profiles in xCell signature: (**A**) lymphoid; and (**B**) myeloid GSEA from xCell signatures, showing immune-cell-enrichment scores. Radial plot highlights GSEA scores for TBI lung versus sham, with red indicating positive, and blue indicating negative, enrichment. Gene sets with significant FDR (*: <0.25, **: <0.05, ***: <0.01) are highlighted and detailed on the right.

In our comprehensive analysis of the impact of traumatic brain injury (TBI) on lung tissue, we employed two sophisticated bioinformatics tools: x-Cell signatures and gene set enrichment analysis (GSEA). x-Cell signatures, a cell-type enrichment analysis based on gene expression data, allow for the deconvolution of complex tissues into their constituent cell types, providing a detailed view of the cellular landscape. This method leverages gene signatures of diverse cell types to estimate their relative abundances in mixed tissue samples, thereby enabling us to dissect the specific contributions of different immune cells in the lung’s response to TBI, as depicted in Figure 3. Utilizing x-Cell signatures, we observed significant alterations in the adaptive immune response, characterized by a pronounced increase in Th1, CD4+, and CD8+ T cells, suggesting their crucial role in the post-TBI pulmonary environment (Figure 3A). Conversely, a notable decrease in memory B cells was observed, pointing to potential suppression or relocation, and highlighting the complex dynamics of the adaptive immune system following TBI. On the innate immune front, our findings, further elucidated through GSEA, revealed a surge in myeloid lineage cells, including monocytes, macrophages, and particularly activated dendritic cells. This underscores their critical role in orchestrating the immune response post-injury (Figure 3B). The analysis also showed a distinctive pattern of dendritic cell behavior, with a decrease in immature dendritic cells alongside an increase in activated forms, suggesting TBI-induced maturation that may influence overall immune modulation in the lung. Additionally, the observed reduction in granulocytes, such as basophils and eosinophils, hints at a selective shift in the lung’s immune response post-TBI, potentially favoring specific immunological pathways over others. These insights into the adaptive and innate immune responses, facilitated by our structured bioinformatics approach using x-Cell signatures and GSEA, underscore the intricate immune modulations occurring in lung tissue after TBI.

### 2.4. Stromal Cells Highlight TBI-Induced Pulmonary Morphogenesis Perturbations with a Notable Emphasis on Fibrosis

**Figure 4:**
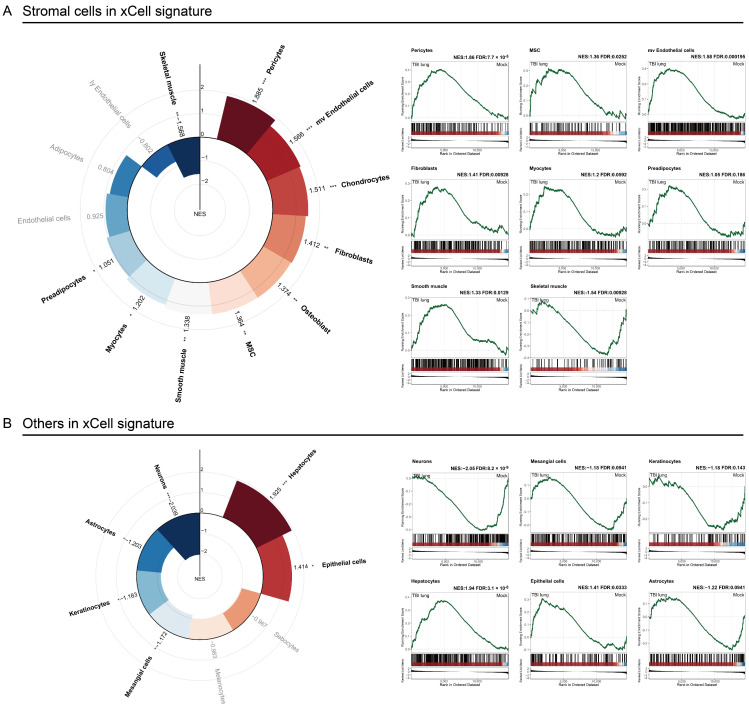
Stromal and other cell-type enrichments in xCell signature post-TBI: (**A**) radial plot of GSEA-enrichment scores for stromal cells from xCell; and (**B**) associated cell types in xCell signature. Comparative GSEA scores between TBI lung and sham are shown, with red bars for positive and blue for negative enrichment. Gene sets with FDR < 0.25 are in bold, with significance levels: *, **, and *** for FDR < 0.25, <0.05, and <0.01, respectively.

A detailed analysis of stromal cell categories post-TBI reveals significant cellular enrichments indicative of complex lung tissue responses. Pericytes, with an NES of 1.86 and FDR < 0.05 (Figure 4A), are highlighted for their essential role in vascular stability and capillary blood flow regulation [33]. Concurrently, the observed increase in microvascular endothelial cells (NES: 1.58, FDR < 0.05) suggests potential disruptions to the blood–air barrier, hinting at altered lung permeability. Fibroblasts (NES: 1.41, FDR < 0.05) and mesenchymal stem cells (MSC) (NES: 1.36, FDR < 0.05) are both enriched, pointing towards the initiation of fibrotic processes and a reparative response to lung damage, respectively [34]. Additionally, the enrichment of smooth muscle cells (NES: 1.33, FDR < 0.05) may signal changes in bronchial dynamics or airway constriction following TBI. Interestingly, a significant decrease in neuronal markers (NES: −2.05, FDR < 0.05) raises questions about neuro-immune interactions and their effects on lung function (Figure 4B). This observation suggests a broader systemic impact of TBI, potentially affecting pulmonary neurology. Collectively, these findings highlight a shift towards fibrosis and vascular remodeling in lung tissue post-TBI, characterized by vascular instability, fibrotic activity, and altered airway dynamics. The noted cellular enrichments underscore the lung’s multifaceted response to injury, including both protective and pathological mechanisms. The decrease in neuronal markers further implicates neuro-immune dynamics as a factor in post-TBI lung physiology, marking an area for future investigation. This comprehensive view of stromal cell responses illuminates the significant, broad impact of TBI on lung tissue, emphasizing the need for targeted research and therapeutic interventions to address fibrosis and vascular remodeling.

### 2.5. Exploring the Systemic Impact of Brain Injury on Lung Associated Cells Using Tabula Sapiens Database

**Figure 5:**
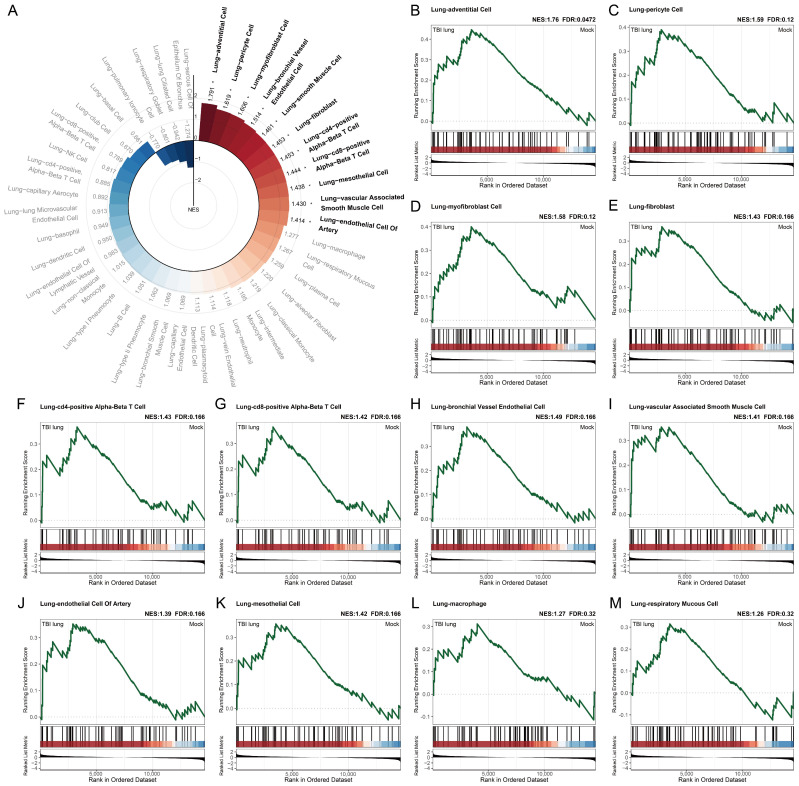
Enrichment of lung-associated cells from Tabula Sapiens via GSEA: (**A**) GSEA of lung-specific single-cell gene signatures from Tabula Sapiens, organized clockwise by NES. Asterisks mark FDR < 0.25 with gene set names beside bars; and (**B**–**M**) top 12 GSEA rankings by NES, contrasting TBI lung group (**left**) with mock group (**right**). Gene sets with FDR < 0.25 are in bold, with significance levels: * for FDR < 0.25.

Our study, employing GSEA on the human single-cell biomarker database, Tabula Sapiens, has uncovered some intriguing findings (Figure 5A). Tabula Sapiens, a preliminary draft of the human cell atlas, comprises nearly 500,000 cells from 24 organs across 15 healthy human subjects, forming an extensive benchmark for our analysis [35]. We focused our study on the cell populations associated with the lungs, as this organ has been understudied in brain-injury-induced immunological responses. Our analysis revealed a pronounced enrichment in lung-adventitial cells, demonstrating the highest positive enrichment score (NES: 1.76) and a false discovery rate (FDR: 0.0472) below the common threshold of 0.05 (Figure 5B). Additionally, lung pericytes showed a notable enrichment (NES: 1.59, FDR: 0.12), suggesting the possibility of an indirect effect on lung vasculature following brain injury (Figure 5C). Further, in our analysis, we detected a significant enrichment in lung myofibroblast cells (NES: 1.58, FDR: 0.12) and lung fibroblasts (NES: 1.43, FDR: 0.166) (Figure 5D,E). These findings might indicate an increased potential for fibroblast differentiation and tissue fibrosis post-brain injury. Additionally, our results suggest infiltration of adaptive immune cells in various organs, including the lungs. This is evident from the positive enrichment of lung CD4-positive alpha–beta T cells and lung CD8-positive alpha–beta T cells, both with an NES of 1.43 and 1.42, and FDR of 0.166 (Figure 5F,G). Our findings also indicate an increase in angiogenesis, as evidenced by the positive enrichment of lung–bronchial vessel endothelial cell, lung–vascular-associated smooth muscle cell, and lung–endothelial cell of the artery (Figure 5H–J). These findings corroborate with the positive enrichment of lung–mesothelial cells (NES: 1.42, FDR: 0.166), potentially linking increased angiogenesis with the inflammatory response induced by brain injury (Figure 5K). Furthermore, while the enrichment scores for lung–macrophage (NES: 1.27, FDR: 0.32) and lung–respiratory mucous cell (NES: 1.26, FDR: 0.32) did not reach the conventional threshold for significance (FDR < 0.25) (Figure 5L,M), these observations still suggest a potential association between brain injury and both the activation of macrophage-related biomarkers and an increase in mucosal cells in lung tissue.

### 2.6. Unraveling the Potential Association between Traumatic Brain Injury and Lung Diseases through GSEA

**Figure 6:**
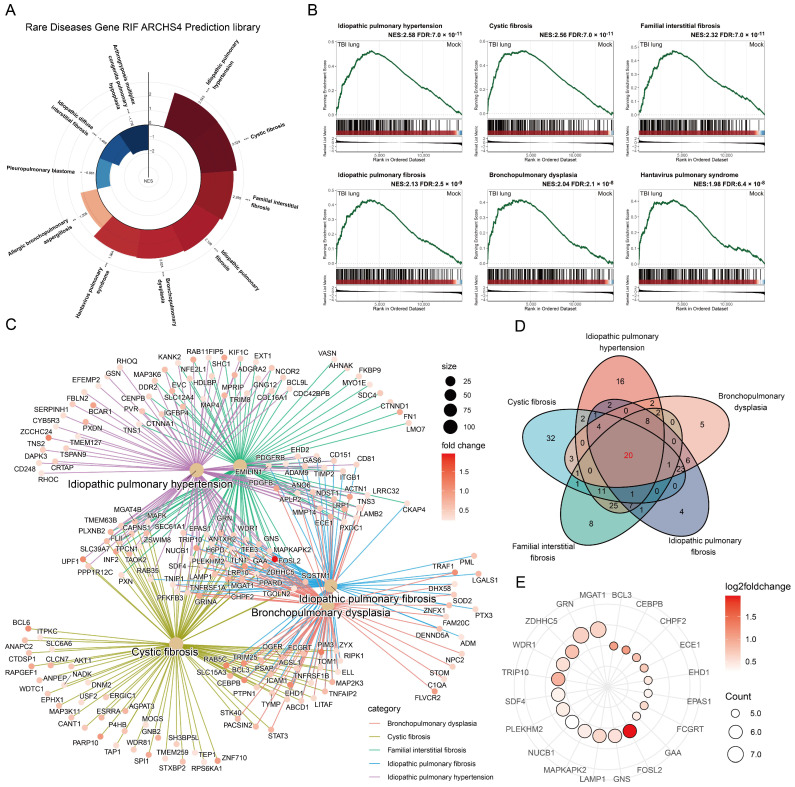
TBI and specific lung diseases: gene set enrichment analysis insights: (**A**) correlation between TBI lung and 10 lung disease gene sets; (**B**) GSEA of top six positively enriched gene sets; (**C**) leading-edge gene associations for top five sets via cnetplot in clusterprofiler; and (**D**) leading-edge gene overlap shown in a Venn diagram. (**E**) Dot plot of top 20 genes by occurrence counts and log2 fold changes. Gene sets with FDR < 0.25 are in bold, with significance levels: *, **, and *** for FDR < 0.25, <0.05, and <0.01, respectively.

In an effort to evaluate the potential implications of TBI on lung pathologies, a comprehensive study was conducted using the Rare Disease Gene RIF ARCHS4 Prediction library. Ten lung disease gene sets were screened for further GSEA, targeting “lung” and “pulmonary” related terms. Although all gene sets exhibited a statistically significant FDR < 0.25, the direction of NES varied, implying a specific spectrum of disease influence due to TBI (Figure 6A). The GSEA results for the top six gene sets positively enriched were shown, with idiopathic pulmonary hypertension leading the charge (NES: 2.58, FDR: 7 × 10^−11^), followed by hepatic pulmonary fibrosis (NES: 2.13, FDR: 1 × 10^−9^), cystic fibrosis (NES: 1.97, FDR: 5 × 10^−8^), familial interstitial fibrosis (NES: 1.88, FDR: 2 × 10^−6^), bronchopulmonary dysplasia (NES: 1.73, FDR: 1 × 10^−4^), and Hantavirus pulmonary syndrome (NES: 1.55, FDR: 3 × 10^−3^) (Figure 6B). The leading-edge genes of the top five gene sets were depicted using the cnetplot of the clusterprofiler, demonstrating considerable overlap (Figure 6C). Further overlap analysis via Venn diagram revealed a common set of 20 genes across these gene sets (Figure 6D). The 20 shared genes, each appearing at least five times in the seven positively enriched lung disease gene sets, were displayed using a dot plot. Among them, *FOSL2* had the greatest log2 fold change and was also listed among the top ten significantly different genes in the volcano plot analysis (Figure 6E). FOSL2, also known as Fra-2, is a basic region–leucine zipper motif transcription factor. Research by UCero et al. found its upregulation to be linked with macrophage-induced idiopathic pulmonary fibrosis, suggesting that TBI might induce pulmonary fibrosis by upregulating FOSL2 and macrophages [36].

### 2.7. Interleukin Family Enrichment Analysis Reveals Key Regulatory Genes in Pulmonary Tissue Post-TBI Using L1000 CRISPR KO Consensus Signature

**Figure 7:**
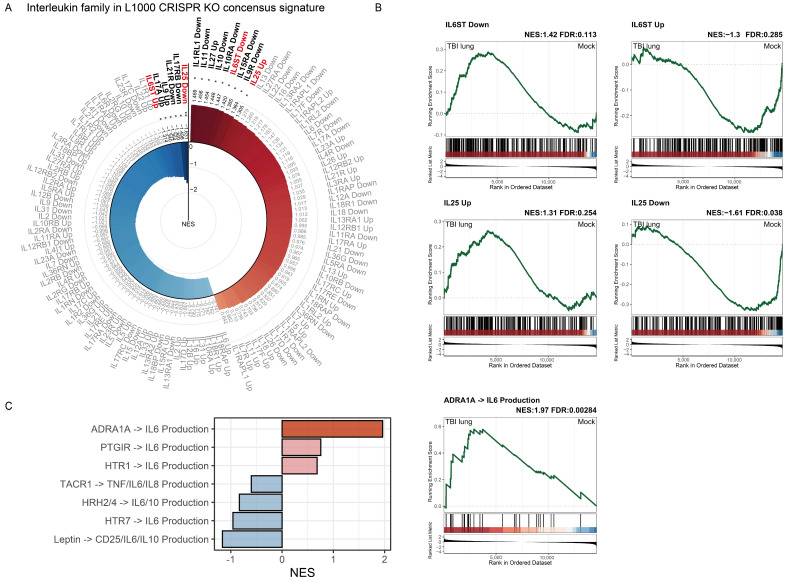
Analysis of the interleukin family in L1000 CRISPR KO consensus signature post-TBI in lung tissue: (**A**) radial plot showcasing the distribution and relationship among various interleukins; (**B**) GSEA plots illustrating the significant enrichment patterns of IL6ST and IL25 after TBI; and (**C**) analysis of IL6-production-associated gene sets derived from the Elsevier Pathway Collection. Bar plot represents the NES with genes exhibiting positive enrichment in red and those with negative enrichment in blue. The corresponding GSEA displays the peak distribution of ADRA1A→IL6 gene sets. Gene sets with FDR < 0.25 are in bold, with significance levels: * and ** for FDR < 0.25 and <0.05, respectively.

Considering the enrichment of immune cells in specific populations and lung tissue following TBI, deciphering the enrichment of the interleukin family can help elucidate the primary underlying mechanisms. We employed the L1000 CRISPR KO consensus signature for our analysis. This database was published by Subramanian et al., meticulously curated to define gene lists upregulated and downregulated upon the deletion of individual genes, resulting in distinct gene sets [37]. Initially, we filtered for interleukin-related gene sets and subsequently performed a GSEA. We stipulated that, for a target to be considered, both its upregulated and downregulated signals must show significant opposite enrichments. Results from the L1000 CRISPR KO consensus signature for the interleukin family are presented in Figure 7A. The following two genes related to the interleukin family met our predefined criteria: *IL6ST* and *IL25.* A positive significant enrichment in *IL6ST* Down combined with a negative significant enrichment in *IL6ST* Up indicates that *IL6ST* plays a pivotal role in the pulmonary tissue effects of TBI (Figure 7B). In contrast, the positive significant enrichment in *IL25* Up combined with a negative significant enrichment in *IL25* Down signifies that the effects of TBI on pulmonary tissues resemble the outcome of *IL25* deletion, suggesting that IL25’s function is likely inhibited (Figure 7B). Further GSEA on the IL6 production gene set (shown in Figure 7C and Figure S1) revealed a significant positive enrichment for *ADRA1A→IL6* Production. This underscores that the generation of IL6 might be attributed to adrenaline-related mechanisms induced by TBI.

### 2.8. Adrenergic Receptor Enrichment and TBI-mediated Pulmonary Impacts in the L1000 Kinase and GPCR Perturbations Database

**Figure 8:**
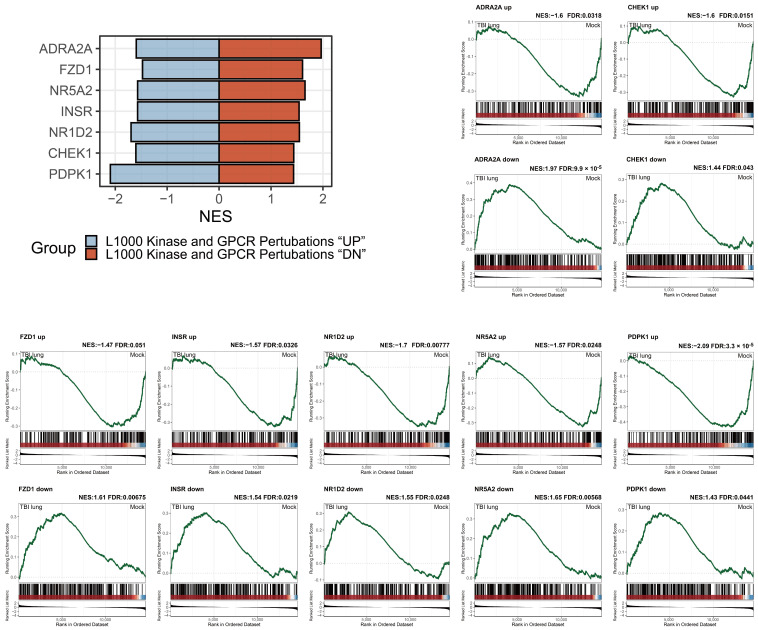
L1000 Kinase and GPCR Perturbations enrichment analysis. Barplot shows NES for genes affecting pulmonary response to TBI, with upregulated genes in blue (Up) and downregulated in red (Dn), filtered by FDR < 0.25. Adjacent GSEA results for gene sets offer additional context.

Utilizing a consistent strategy, we probed the L1000 Kinase and G Protein-Coupled Receptor (GPCR) Perturbations database to discern genes potentially implicated in the pulmonary impacts attributed to TBI. As per the definitions set by L1000 Kinase and GPCR Perturbations, gene sets categorized as “UP” represent genes upregulated upon perturbation, while those under “DN” denote genes downregulated upon such disturbances. Consequently, we adopted a bidirectional enrichment criterion with contrasting positive and negative indications as our screening parameter. Results presented in Figure 8 highlight *ADRA2A* as being bidirectionally, significantly enriched. This finding is in consonance with the *ADRA1A* result from Figure 7, underscoring the probable importance of adrenergic receptors in TBI-mediated pulmonary effects. Other genes emerging from this analysis include *FZD1*, *NR5A2*, *INSR*, *NR1D2*, *CHEK1*, and *PDPK1*. These genes, collectively, suggest a complex molecular landscape associated with TBI’s pulmonary consequences.

## 3. Discussion

Traumatic brain injury is increasingly recognized as an instigator of systemic responses, with effects manifesting in organs distant from the primary site of injury [9]. The lungs, given their extensive vasculature and intimate connection with systemic circulation, become particularly vulnerable to such systemic repercussions. Our research focused on dissecting the molecular and cellular dynamics of lung tissues in the aftermath of TBI. The underpinnings of inflammatory cascades in TBI-induced lung pathologies are paramount. Our study, particularly Figure 1C, revealed an upregulated transcriptional profile of genes associated with inflammatory responses. The enhancement in genes related to the “positive regulation of interleukin-4 production” can be perceived as the lung’s defensive maneuver, pivoting towards an anti-inflammatory stance. IL-4’s role in mediating lung defense mechanisms against injury has been previously reported on, buttressing our findings [38]. In contrast, genes governing neurotransmitter dynamics, notably “dopamine secretion”, “neurotransmitter receptor activity”, and others, exhibited downregulation. This highlights the dual impact of TBI in promoting inflammatory responses while potentially disrupting neural signaling, which may indirectly affect lung tissues. This decrement, although seemingly counterintuitive, can be comprehended within the framework of a dysregulated neuro-respiratory inflammasome axis—an observation previously documented in [30].

Macrophages, widely acknowledged as sentinel cells in tissue homeostasis, appear to be significantly entwined in the post-TBI pulmonary milieu. Our findings corroborate the research conducted by Henry et al., which identified pronounced pulmonary inflammation characterized by increased neutrophil levels, the accumulation of activated alveolar macrophages, and heightened pulmonary LTB4 production 24 h post-injury. However, our research goes a step further by uncovering a distinct relationship between macrophages and the *C/EBPβ*→*NF-kB* signaling pathway, as elucidated in Figure 2B. C/EBPβ, a transcription factor of immense physiological relevance, has been previously implicated in acute lung injuries, especially those triggered by immune complexes [32]. The contribution of C/EBPα and C/EBPβ in modulating the differentiation of airway epithelial cells further consolidates their indispensable role during lung organogenesis [39]. Delving deeper into the cellular contours, the portrayal of immune cell populations post-TBI appeared to be a mosaic of varied responses. As shown in our results, Th1 cells, CD4, and CD8 effector memory cells were positively enriched. This aligns with the documented lung injury attributed to alloreactive Th1 cells, underscoring their contributory role in tissue pathology [40]. B cells, especially memory B cells, curiously emerged with a negative enrichment. Their established protective demeanor in sterile particulate-induced lung injury makes this finding noteworthy, suggesting a potential imbalance in B cell dynamics post-TBI [41]. In the broader cellular theater, monocytes, macrophages, and activated dendritic cells etched their significance with positive enrichment profiles, echoing their acknowledged roles in various lung injury paradigms [42,43].

Figure 4 and Figure 5 unravel the enrichment dynamics of stromal cells and other cellular subtypes. The spotlight on neurons, given their negative enrichment, is an intriguing facet, potentially hinting at compromised neuro-immune interplay [30]. The propensity of the lung towards fibrotic pathways post-TBI was accentuated by our observations in Figure 6 and Figure 7. Conditions typified by fibrosis, such as cystic fibrosis and idiopathic pulmonary fibrosis, emerged with positive enrichments. Recent studies by Saef et al. have indicated that TBI of any severity is linked to an increased risk of chronic cardiovascular, endocrine, and neurological comorbidities in patients without pre-existing conditions [44]. Our results suggest that pulmonary fibrosis could also be considered for targeted screening for multisystem diseases following TBI. Notably, the gene *FOSL2* was also prominently enriched in our dataset. *FOSL2*, also known as *Fra-2*, is a critical gene within the Fos family that acts as a novel mediator of cell proliferation, differentiation, and transformation in the fibrotic pathophysiology of certain conditions, including scleroderma and pulmonary hypertension [45]. Previous research has demonstrated an increase in FOSL2 levels in cases of ventilator-induced lung injury [46]. FOSL2 has also been implicated in pulmonary fibrosis, with its overexpression in macrophages promoting fibrotic changes in the lung [36]. Its role in our context suggests a potential molecular link between TBI and ensuing fibrotic alterations in the lung.

*IL6ST* and *IL25*, with their polarized enrichment patterns, contributed another layer to this narrative, emphasizing the interplay between inflammation and fibrosis [47,48]. In TBI, increased IL-6 levels are linked to poorer outcomes, yet the function of IL-6 in injury response is paradoxical. While IL-6 enhances neurogenesis and wound healing in TBI animal models, it can also lead to breakdowns in the blood–brain barrier (BBB) and exacerbate cerebral edema [49]. In addition, increased levels of IL-6 in the plasma are linked to the onset of ARDS in patients suffering from severe TBI [50]. Some potential candidates for systemic therapy in TBI have also demonstrated attenuation of TBI-induced ALI, accompanied by reduced mRNA levels of IL-6 in the lung [25,26]. Our results also demonstrated that the generation of *IL6* might be attributed to mechanisms related to α1-adrenergic receptor stimulation induced by TBI (Figure 7C), which correlates with previous studies [51].

Conversely, neurogenic pulmonary edema is a consequence that often accompanies severe neurological injury. The presence of neurogenic pulmonary edema in patients with brain injuries is linked to a grim prognosis [52]. Sympathetic activation plays a crucial role in the development of neurogenic pulmonary edema [53]. Figure 7 and Figure 8 underscore the potential significance of adrenergic receptors, particularly ADRA1A and ADRA2A, in shaping the pulmonary response following TBI [54]. This finding aligns with previous studies indicating that sympathetic activation following TBI, leading to pulmonary edema, may be prevented using α-adrenergic receptor antagonists [55]. Various genes, notably *FZD1*, *NR5A2*, and *CHEK1*, converged on the theme of idiopathic pulmonary fibrosis, reinforcing the fibrotic narrative post-TBI [56,57]. In the cascade of genes that emerged having been significantly altered post-TBI, two genes, *NR1D2* and *CHEK1*, drew particular attention. *NR1D2*, a key component in the circadian rhythm molecular clock, was underscored in our findings. Its dysregulation could potentially hint at a disorganized lung tissue homeostasis in the wake of TBI. Intriguingly, *NR1D2* has also been associated with lung fibrotic progression, specifically in the context of collagen stabilization [58]. Such a connection suggests a temporally regulated collagen stabilization mechanism in the lung, which might be perturbed following TBI. *CHEK1*, on the other hand, emerges in the panorama of cell cycle checkpoint regulation. Its aberrant expression is suggestive of a compromised cellular DNA damage response in lung tissues. Moreover, a deeper dive into previous reports reveals *CHEK1*’s involvement in the pathogenesis of idiopathic pulmonary fibrosis, portraying it as a critical player in this fibrotic milieu [57]. *CHEK1*’s dysregulation post-TBI, hence, opens the window to the possibility of uncontrolled cell cycle progression, leading to fibrotic predispositions. *PDPK1* emerged as a gene of interest, having been previously documented in the context of acute lung injury, especially with its potential in modulating the NF-κB/p65 signaling pathway [59].

## 4. Materials and Methods

### 4.1. Traumatic Brain Injury Animal Model

In our study, we employed a unilateral controlled cortical impact as the TBI model on adult C57BL/6 male mice, aged 7 weeks, following the methodology adapted from our previous publication [29]. In brief, the mice were anesthetized with isoflurane and fixed in a stereotaxic frame, with a topical anesthetic applied to the surgical area to reduce discomfort. During the procedure, body temperature was maintained using a warming pad, and anesthesia depth was periodically checked using the toe-pinch method. A 4 mm diameter craniotomy was performed on the right parietal temporal cortex. In the experimental paradigm, the TBI cohort comprised five murine subjects, subjected to a meticulously calibrated cortical insult via an impactor device. Conversely, the sham cohort, designed to serve as a procedural control, initially encompassed four subjects, each undergoing a craniotomy devoid of subsequent cortical trauma. Notably, the sham cohort’s numerical discrepancy, in comparison with the TBI cohort, emanated from an unforeseen complication in sample procurement, culminating in the attrition of one specimen from the control ensemble. Post-operative care included rehydration with warm saline and temperature maintenance.

Following euthanasia via isoflurane and confirmation of death through respiratory cessation, mice were perfused with PBS, and lung tissues were harvested. The tissues were minced and homogenized using a tissue homogenizer with zirconium oxide beads in TRIzol Reagent (Thermo Fisher Scientific, Waltham, MA, USA) for RNA extraction and stored at −80 °C until further analysis. All procedures were conducted in compliance with the ethical standards of the National Defense Medical Center’s Laboratory Animal Center, accredited by the Association for Assessment and Accreditation of Laboratory Animal Care (AAALAC). Lung tissue samples were collected under approved protocols.

### 4.2. mRNA Expression Profiling

Mouse lung tissues were processed, and total RNA was extracted using TRIzol reagent, following the manufacturer’s guidelines. This methodology was adapted from our previous publication, with modifications for lung tissue analysis, and updated to reflect the use of R version 4.3.2 for subsequent analyses [29]. For details on monitoring RNA quality, please refer to our previous publication.

### 4.3. Differential Expression Analysis

Analyses were conducted using RStudio (version 2023.12.1 Build 443) and R (version 4.3.2). The DEGs between groups was examined using the EdgeR package (version 4.0.16) [60]. The analysis resulted in a data frame containing gene symbols, log2 fold changes, and FDR, which were designated as a genelist in R. For detailed raw data comparing TBI-affected lung tissues to control groups, refer to Table S1. The Benjamin–Hochberg procedure was applied to adjust enriched *p*-values for statistical significance assessment, with genes deemed differentially expressed at a |log2 fold change| ≥ 1 and a FDR < 0.05.

### 4.4. Enrichment Analysis

To comprehensively represent the biological processes most affected by TBI in lung tissues, extensive differential gene expression over representative analysis (ORA) and gene set enrichment analyses were performed. ORA and GSEA offer complementary insights into the biological significance of our data. ORA’s strength lies in its ability to detect over-represented pathways or functions in a predefined gene set, which is particularly useful for understanding the specific biological themes present in our data. However, ORA requires a pre-selected list of genes, which might introduce bias by excluding genes based on arbitrary cutoffs. GSEA addresses this limitation by analyzing the entire ranked list of genes without pre-filtering, ensuring that the analysis considers the subtle expression changes across all genes. This comprehensive approach reduces the risk of overlooking potentially relevant biological signals due to stringent selection criteria, offering a more inclusive and unbiased view of the gene expression landscape. Volcano plots were generated using the ggplot2 package (version 3.4.0), highlighting the top 30 genes with the smallest FDR and sorted by fold change values. ClueGO (version 2.5.10) in Cytoscape (version 3.9.1) was utilized for visualization [61], with GOBP enrichment analysis conducted using the “c5.go.bp.v7.5.1.symbols.gmt” file from MSigDB (https://www.gsea-msigdb.org/gsea/msigdb/collections.jsp, accessed on 8 March 2023), applying filters for “FDR cutoff < 0.05” and “Edge cutoff > 0.375” [62,63]. The GOcircle function of the GOplot (version 1.0.2) was used to display individual gene sets, including fold changes of genes and overall enrichment (Z-score) [64]. The DEG list was further analyzed using the gseaplot2 function of the enrichplot (version 1.22.0) for GSEA plots, with related gene sets downloaded from the EnrichR website (https://maayanlab.cloud/Enrichr/, accessed on 15 July 2023) [65,66]. EnrichR is a comprehensive gene set enrichment analysis web tool that enables researchers to analyze and visualize gene lists for enriched terms. This analysis yielded a data frame comprising NES and FDR, which facilitated a deeper understanding of the underlying biological processes and pathways influenced by the differential gene expression observed in our study. The cnetplot, heatplot, and Upset plot functions from the clusterProfiler (version 4.10.0), an R package widely used for statistical analysis and visualization of functional profiles for genes and gene clusters, were employed to examine the overlap among genes within related gene sets. For x-Cell signature analysis, three source cell-related gene sets provided by Aran et al. were integrated and analyzed using the GSEA function of clusterProfiler [67,68], with results visualized as radial plots in ggplot2, displaying GSEA enrichment for each cell type individually. The Rare Disease Gene RIF ARCHS4 Prediction library, downloaded from EnrichR, was used with clusterProfiler’s GSEA and cnetplot function to present genes matching different disease-related gene sets, with common genes visualized using ggVennDiagram package (version 1.5.2). The L1000 CRISPR KO consensus signature and 1000 Kinase and GPCR Perturbations, obtained from the SigCom LINCS website (https://maayanlab.cloud/sigcom-lincs/#/About, accessed on 25 July 2023) [37], represent an advanced online platform designed to explore and interrogate the Library of Integrated Network-Based Cellular Signatures (LINCS) data. These were analyzed using the clusterProfiler’s GSEA function to quantify the NES, filtering individual gene sets with opposing UP and DN NES that were both significantly enriched.

### 4.5. Visualization Techniques

For the visualization of the GSEA results, we employed the ggplot2 package (version 3.4.0), a versatile tool in R for creating intricate and customizable plots. We specifically focused on creating bar plots to represent the NES and FDR values, which provided a clear and concise overview of the gene sets significantly enriched in our dataset. To enhance the readability and interpretability of these plots, we transformed them into radial (polar) plots using the coord_polar() function in ggplot2. This transformation arranged the bar plots radially, simplifying the comparison and contrast of enrichment scores across various gene sets and providing a more intuitive understanding of the data.

## 5. Conclusions

Our investigation into the systemic effects of TBI on lung tissues, through transcriptomic analyses, elucidates pivotal alterations in gene expression, immune modulation, and signaling pathways. We unveiled a significant activation of the *C/EBPβ*→*NF-kB* pathway, instrumental in macrophage activation and inflammatory responses, alongside notable immune cell alterations. Elevated *IL-6* levels further underscore the pathway’s dual role in both facilitating recovery and engendering post-TBI complications. The identification of adrenergic receptors also highlights their importance in mediating pulmonary responses to TBI. Our findings preliminarily suggest that TBI might precipitate fibrotic changes in lung tissues, a phenomenon necessitating extensive investigation due to its profound implications for patient recuperation and therapeutic intervention.

It is imperative, however, to acknowledge the inherent limitations associated with transcriptomic analyses. Such limitations include the potential for variability in gene expression not necessarily reflecting protein levels or functional changes in the tissue, the challenge of distinguishing direct effects of TBI from secondary systemic responses, and the need for rigorous validation of identified pathways through complementary techniques. Furthermore, the complexity of the TBI-induced systemic response, encompassing a multitude of interacting pathways and cell types, may not be fully captured by transcriptomic snapshots. Despite these constraints, our research lays foundational groundwork for future therapeutic strategies aimed at ameliorating the pulmonary sequelae of TBI, with an emphasis on counteracting inflammation, fibrosis, and the overarching systemic repercussions of brain injuries.

## Data Availability

The original contributions presented in the study are included in the Supplementary Table S1. Further inquiries can be directed to the corresponding author. The data are not publicly available because they are part of an ongoing study.

## Supplementary Materials

The following supporting information can be downloaded at: https://www.mdpi.com/article/10.3390/ijms25053018/s1.

## Abbreviations

TBI: traumatic brain injury; ALI: acute lung injury; ARDS: acute respiratory distress syndrome; DEG: differentially expressed genes; FDR: false discovery rate; C/EBPβ: CCAAT/enhancer binding protein beta; NES: normalized enrichment score; GSEA: gene set enrichment analysis; MSC: mesenchymal stem cells; GPCR: G protein-coupled receptor; BBB: blood–brain barrier; AAALAC: Association for Assessment and Accreditation of Laboratory Animal Care; ORA: over representative analysis; LINCS: Library of Integrated Network-based Cellular Signatures.
