# Supplementary material for: Pulmonary Effects of Traumatic Brain Injury in Mice: A Gene Set Enrichment Analysis
Source: Int J Mol Sci. 2024 Mar 5;25(5):3018. doi: 10.3390/ijms25053018 (PMC10931562; doi:10.3390/ijms25053018)
Supplement: Supplementary file 1 [file ijms-25-03018-s001.zip › Supplementary figure 1.pdf]

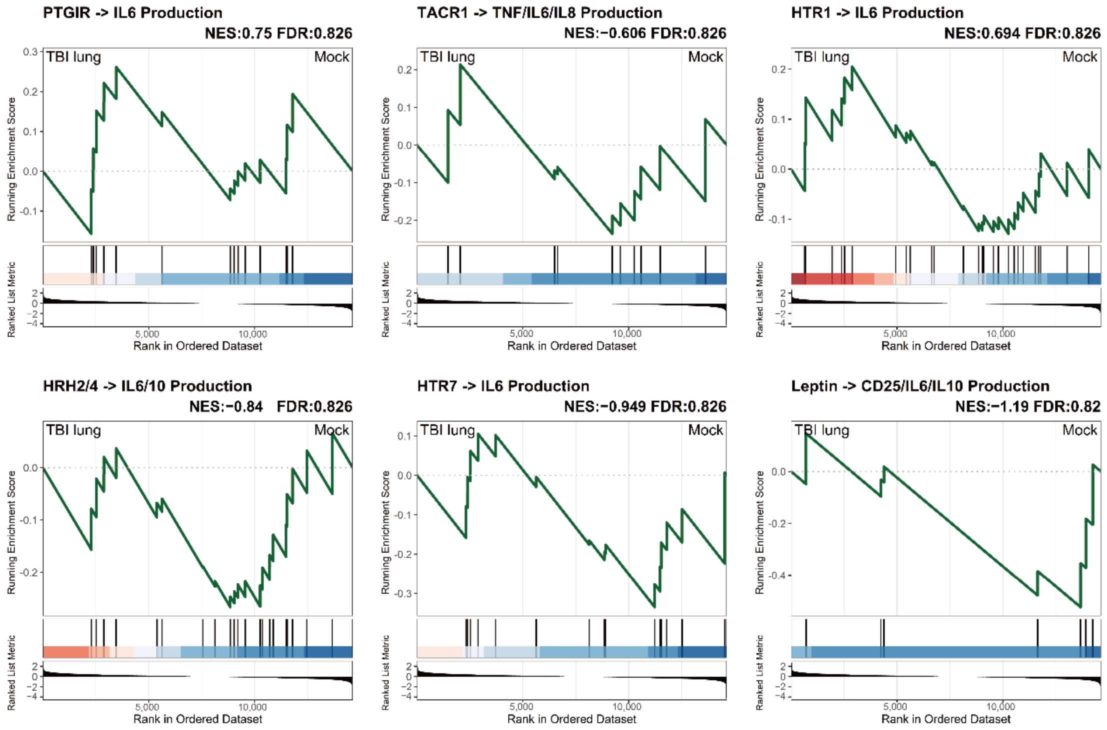

Supplementary figure 1. Analysis of IL6 production-associated gene sets derived from the Elsevier Pathway Collection. The corresponding GSEA displays the peak distribution of individual gene sets.
